# Polyethylene glycol-mediated fusion of herpes simplex type 1 virions with the plasma membrane of cells that support endocytic entry

**DOI:** 10.1186/s12985-015-0423-0

**Published:** 2015-11-16

**Authors:** Erik B. Walker, Suzanne M. Pritchard, Cristina W. Cunha, Hector C. Aguilar, Anthony V. Nicola

**Affiliations:** Department of Veterinary Microbiology and Pathology, College of Veterinary Medicine, Washington State University, Pullman, WA 99164 USA; Paul G. Allen School for Global Animal Health, Washington State University, Pullman, WA 99164 USA

**Keywords:** Herpesviruses, Herpes simplex virus, Viral entry, Virus-cell fusion, Membrane fusion, Endocytosis, Receptors, Low pH

## Abstract

**Background:**

Mouse B78 cells and Chinese hamster ovary (CHO) cells are important to the study of HSV-1 entry because both are resistant to infection at the level of viral entry. When provided with a gD-receptor such as nectin-1, these cells support HSV-1 entry by an endocytosis pathway. Treating some viruses bound to cells with the fusogen polyethylene glycol (PEG) mediates viral fusion with the cell surface but is insufficient to rescue viral entry. It is unclear whether PEG-mediated fusion of HSV with the plasma membrane of B78 or CHO cells results in successful entry and infection.

**Findings:**

Treating HSV-1 bound to B78 or CHO cells with PEG allowed viral entry as measured by virus-induced beta-galactosidase activity. Based on the mechanism of PEG action, we propose that entry likely proceeds by direct fusion of HSV particles with the plasma membrane. Under the conditions tested, PEG-mediated infection of CHO cells progressed to the level of HSV late gene expression, while B78 cells supported HSV DNA replication. We tested whether proteolysis or acidification of cell-bound virions could trigger HSV fusion with the plasma membrane. Under the conditions tested, mildly acidic pH of 5–6 or the protease trypsin were not capable of triggering HSV-1 fusion as compared to PEG-treated cell-bound virions.

**Conclusions:**

B78 cells and CHO cells, which typically endocytose HSV prior to viral penetration, are capable of supporting HSV-1 entry via direct penetration. HSV capsids delivered directly to the cytosol at the periphery of these cells complete the entry process. B78 and CHO cells may be utilized to screen for factors that trigger entry as a consequence of fusion of virions with the cell surface, and PEG treatment can provide a necessary control.

## Background

To accomplish entry via virus-cell membrane fusion, viruses depend on one or more cellular triggers such as endosomal pH, receptor engagement, and/or enzymatic cleavage [1–6]. The host requirements for herpesviral fusion and entry are incompletely defined. Herpes simplex virus (HSV) binds to a cellular receptor such as nectin-1 or HVEM. This interaction is essential, but it is not clear if it is sufficient for fusion and entry [7, 8]. There is mounting evidence for additional cellular cues for HSV entry such as intracellular low pH [9–14] and receptors that bind either gB [15–17] or gH [18–21].

HSV is able to traverse a pH-dependent, endocytic pathway [9]. The endosomal route is utilized in many cell lines including the model cell lines, CHO-nectin-1 and B78-nectin-1 [9, 10, 12, 22–25]. HSV entry into several human epithelial lines including epidermal keratinocytes occurs via a low pH-dependent pathway similar to CHO-receptor cells [9, 10, 21, 25–27]. Entry into human neuronal SY5Y cells and human epidermal cell line A431 proceeds via a pathway similar to that in B78-receptor cells [10, 24]. In addition, HSV enters several human neuronal cell types and model Vero cells by direct, pH-independent penetration at the plasma membrane [10, 28–31]. An emerging theme for herpesvirus entry is the utilization of endocytic and non-endocytic routes in a cell type-dependent manner [9, 10, 32–37].

Receptor-deficient cells have been valuable tools for understanding HSV entry. For example, the screening of expression libraries in CHO cells was a key approach in the identification of several cellular entry factors and receptors for HSV [15, 38–41]. B78 and CHO cells are both resistant to HSV-1 entry [42, 43]. However, when provided with a gD-receptor such as nectin-1, these cells support HSV entry by an endocytosis pathway.

The full complement of cellular factors that are sufficient for HSV entry is not clear. Receptor-negative cells that normally support entry by endocytosis are excellent tools to screen for factors that trigger HSV-cell fusion. In order for such a system to be useful, entry that results from fusion with the plasma membrane must be detectable. Since HSV entry pathway varies with cell type, it is possible that cells that typically support HSV capsid release and penetration from an endocytic compartment may not properly target a capsid that appears in the cell periphery. In fact, Semliki Forest Virus, which normally enters by endocytosis, can be artificially fused with the plasma membrane of CHO cells as detected by electron microscopy. However, the de-enveloped SFV capsid remains trapped in the cell periphery and complete entry and infection does not occur [44]. The chemical fusogen, polyethylene glycol (PEG) has been utilized to artificially fuse HSV with cells to analyze envelope glycoprotein function [45–47]. PEG has been underutilized as a means to address cellular factors involved in HSV entry and fusion. Here, we use PEG-mediated fusion to determine that HSV receptor-negative B78 and CHO cells are able to support the plasma membrane route of entry. We then use this model system to evaluate the ability of a protease and low pH to trigger virus-cell fusion.

## Results and discussion

B78H1 Gal11 cells (referred to as B78 cells in this study) are mouse melanoma cells derived from parental B78H1 cells, which are resistant to HSV entry. B78 cells are stably transformed with the *Escherichia coli lacZ* gene under the control of the HSV ICP4 promoter. B78C10 cells (referred to as B78-nectin-1 cells in this study) stably express the *lacZ* gene and the human gD-receptor nectin-1. Nectin-1 renders B78 cells susceptible to HSV entry. CHO-IEβ8 cells are a hamster cell line derived from the parental CHO-K1 cell line and are stably transformed with the *Escherichia coli lacZ* gene under the control of the HSV ICP4 promoter. CHO-nectin-1 (M3A) cells are CHO-IEβ8 cells stably transformed with the human nectin-1 gene. The beta-galactosidase reporter assay is widely used as an indicator of HSV entry.

**Fig. 1:**
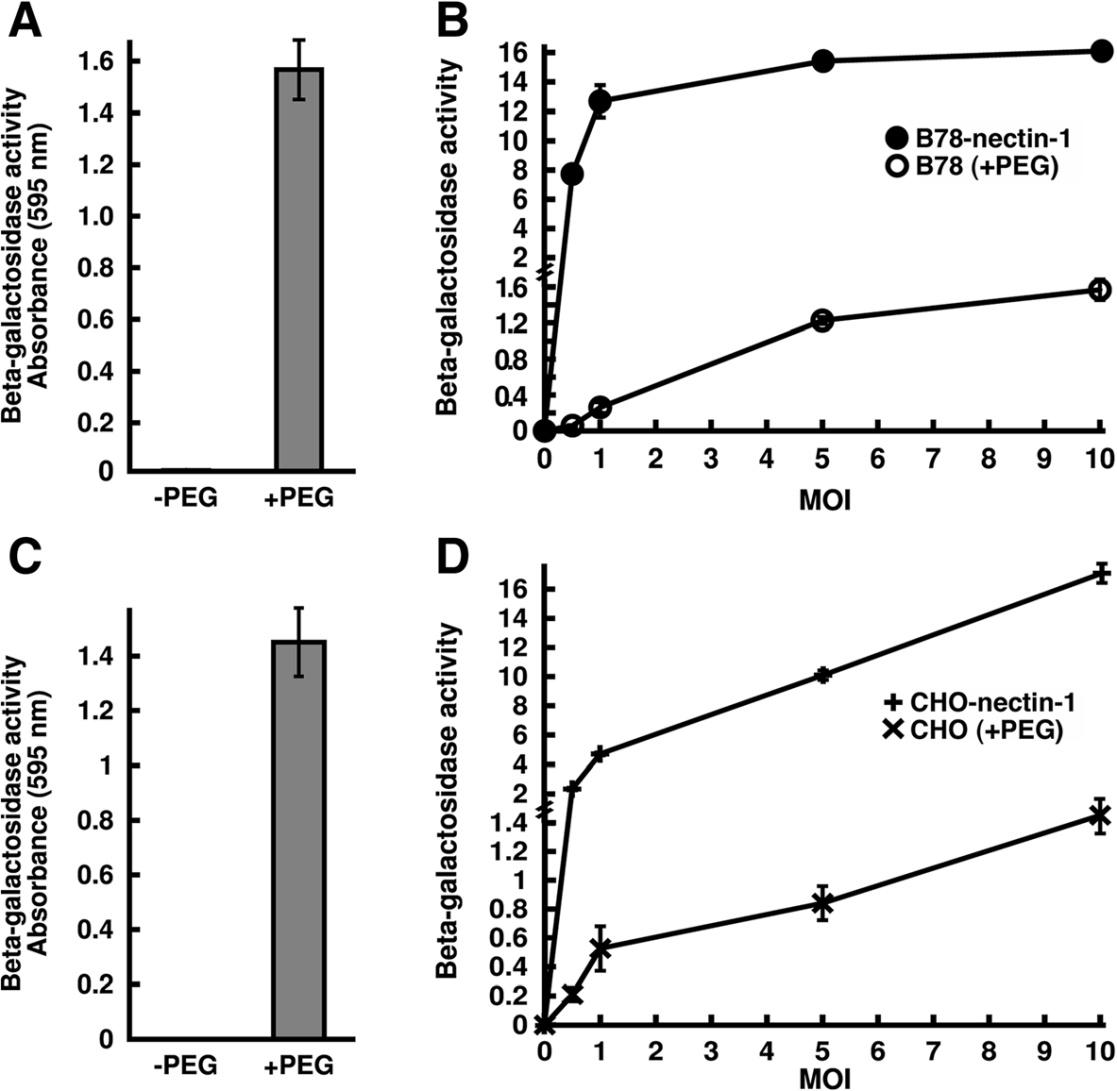
PEG-mediated entry of HSV-1 into cells that are resistant to entry and that normally mediate entry via an endocytosis pathway. HSV-1 strain KOS was bound to B78 cells (**a**) or CHO cells (**c**) (MOI of 10) for 2 h at 4 °C. Cell bound virus was treated with PEG 6000 (+ PEG) or mock-treated (− PEG). At 7 h post-infection, beta-galactosidase activity was detected as an indication of viral entry [74]. **b**, **d** HSV-1 KOS was bound to B78 and B78-nectin-1 cells (**b**) or CHO and CHO-nectin-1 cells (**d**) for 2 h at 4 °C at the indicated MOIs ranging from 0.5 to 10. B78 and CHO cell samples were treated with PEG and B78-nectin-1 and CHO-nectin-1 samples were mock treated. Beta-galactosidase activity was measured at 7 h p.i. **b**, **d** To allow a more direct comparison between samples of disparate reactivity, a fraction of the B78-nectin-1 and CHO-nectin-1 samples was measured. Values shown represent the beta-galactosidase activity from equivalent cell numbers. Each value is the mean of quadruplicate determinations with standard deviation. One representative experiment of at least three independent experiments is shown

As expected [42, 43], adding HSV-1 at an MOI of 10 to wild type B78 or CHO cells did not result in detectable viral entry (Fig. 1a and c) as measured by beta-galactosidase activity. PEG 6000 solid (Sigma) was melted in an Amsco autoclave (STERIS, Mentor, OH). A 1 g/ml solution of PEG 6000 in serum-free cell culture medium was incubated at 37 °C until dissolved. Ice-cold HSV-1 strain KOS was added to confluent cell monolayers (MOI of 10) for 2 h at 4 °C to allow for cell binding. Cultures were washed with warm PBS. Cell-bound virus was treated with PEG 6000 or mock-treated. PEG solution was added to the cell surface for 30 s, and then cells were rinsed thrice with warm PBS. Complete cell culture medium was then added to cells. When PEG 6000 was added to HSV-1 bound to the surface of either B78 or CHO cells, viral entry was detected as determined by beta-galactosidase activity (Fig. 1a and c). PEG has also been shown to mediate infection of swine testis (ST) cells, which are resistant to HSV-1 entry [48]. The mode of HSV-1 entry into ST cells has not been established. In the B78 and CHO cells used in this study, following membrane fusion, tegument VP16 is translocated to the nucleus and induces beta-galactosidase expression driven by the ICP4 promoter. These results suggest that PEG successfully triggered fusion between the viral envelope and the plasma membrane. When an appropriate gD-receptor is present, the subcellular site of fusion in both B78 and CHO cells is an internal, endosomal membrane. Thus, if appropriate conditions are provided, HSV-1 can fuse with the plasma membrane of cells that typically support viral endocytosis. This fusion can be detected by downstream reporter gene expression.

If the enhanced beta-galactosidase signal in the PEG-treated samples (Fig. 1a) is due to HSV-1 entry, then adding increasing amounts of HSV-1 should result in a concomitant increase in reporter expression. Indeed, beta-galactosidase activity increased in a virus dose-dependent manner when MOIs ranging from 0 to 10 were tested in the PEG experiment on wild type B78 and CHO cells (Fig. 1b and d). Importantly, PEG treatment of CHO or B78 cells in the absence of virus did not result in beta-galactosidase activity above background (MOI of 0 in Fig. 1b and d). To our knowledge, this is the first demonstration of PEG rescuing HSV infection of receptor-negative CHO and B78 cells. PEG has been used previously to rescue the fusion activity of fusion-dead virus mutants [45–47]. We gauged the efficiency of PEG-triggered entry as compared to gD-receptor-mediated entry via endocytosis. In general, PEG-mediated entry into the receptor-negative cells was less efficient than entry into the corresponding cells expressing the gD-receptor nectin-1 (Fig. 1b and d). At all MOIs tested for CHO cells and at the higher MOIs of 5 and 10 for the B78 cells, the nectin-1 expressing cells supported entry ~ 9 to 12-times more effectively than PEG-induced fusion in the respective receptor-negative cells. Furthermore, PEG-mediated entry into B78 cells at the lowest MOIs tested (0.5 and 1) was ~ 40 to 70 times less efficient than entry into B78-nectin-1 cells (Fig. 1b). These differences may be even greater when comparing fully entry-competent viral samples (if these could be obtained), as it is possible that PEG fusion might permit infection by virions that would otherwise be defective for a receptor-dependent entry pathway. In contrast to HSV-1, HSV-2 strains successfully enter wild type CHO cells [41, 42], so it is of future interest to determine the effect of PEG treatment on HSV-2.

**Fig. 2:**
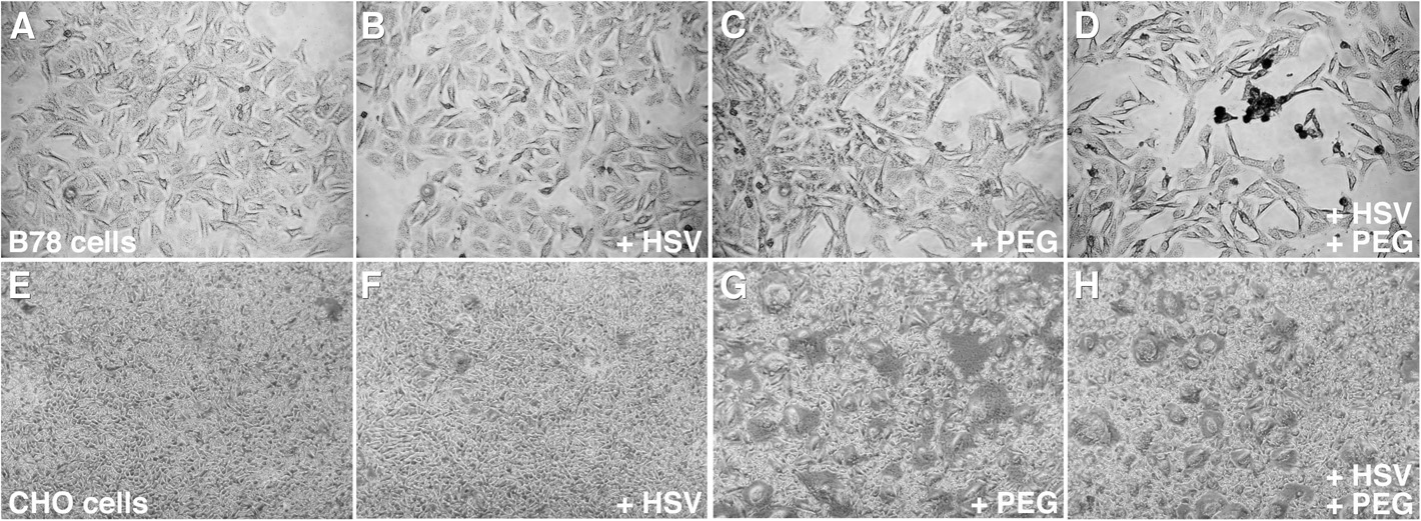
Microscopic analysis of the effect of PEG 6000 on HSV-1 infection of receptor-negative cells. B78 cells (**a**-**d**) or CHO cells (**e**-**h**) were infected with HSV-1 strain KOS (MOI of 10) (**b**, **d**, **f**, **h**) or remained uninfected (**a**, **c**, **e**, **g**) for 2 h at 4 °C. Cultures were treated with PEG 6000 (**c**, **d**, **g**, **h**) or mock-treated (**a**, **b**, **e**, **f**). At 24 h post-infection (p.i.), cultures were fixed with ice-cold methanol-acetone solution (2:1 ratio) for 20 min at −20 °C and air dried. HSV antigen-positive cells were detected by immunoperoxidase staining with anti-HSV polyclonal antibody HR50 (Fitzgerald Industries, Concord, MA) [75]. Magnification, 5X

We microscopically examined the effect of PEG treatment on HSV infectivity of receptor-negative cells. As expected, addition of HSV-1 alone to wild type B78 or CHO cells resulted in no detectable infected cells (Fig. 2b and f), virtually indistinguishable from uninfected cells (Fig. 2a and e). This is consistent with the resistance of B78 cells and CHO cells to HSV-1 entry. PEG treatment of HSV-1 bound to B78 cells followed by 24 h infection resulted in HSV-antigen positive, single cells and small clusters of cells, as detected by immunoperoxidase staining with an anti-HSV polyclonal antibody (Fig. 2d). This is visual evidence that PEG mediates HSV entry into receptor-negative B78 cells. Treatment with PEG alone did not result in HSV antigen staining in this assay (Fig. 2c). HSV forms large plaques comprised of dozens of infected cells on B78 cells that express gD-receptors such as nectin-1 and HVEM, consistent with a gD-receptor allowing entry [43]. The single-infected cells and small plaques in the PEG treated samples (Fig. 2d) are consistent with the requirement of gD-receptors to mediate spread between B78 cells [49].

In contrast to the B78 cell result, PEG treatment of HSV-1 bound to CHO cells followed by 24 h infection did not result in detection of HSV-antigen positive cells (Fig. 2h). Thus, although PEG mediates entry of HSV-1 into receptor-negative CHO cells as measured by reporter gene expression (Fig. 1), synthesis of viral antigens is not detected by immunoperoxidase staining of fixed cells at 24 h. CHO cells that express gD-receptors do not support wild type HSV-1 plaque formation [39, 42]. Together with Fig. 1c and d, these results support the notion that beta-galactosidase reporter gene expression is more sensitive for detecting entry into CHO cells than is staining cells for HSV-specific antigen. Interestingly, treatment of CHO cells with PEG alone caused noticeable cell-cell fusion (Fig. 2g). Similar fusion was not detected with B78 cells (Fig. 2c). That CHO cells are prone to fusion is important for interpreting results from HSV cell-cell fusion assays, in which CHO cells have been widely used [50–53]. In fact, when CHO cell preparations are mixed in fusion assays the background fusion can be high ([50]; data not shown).

**Fig. 3:**
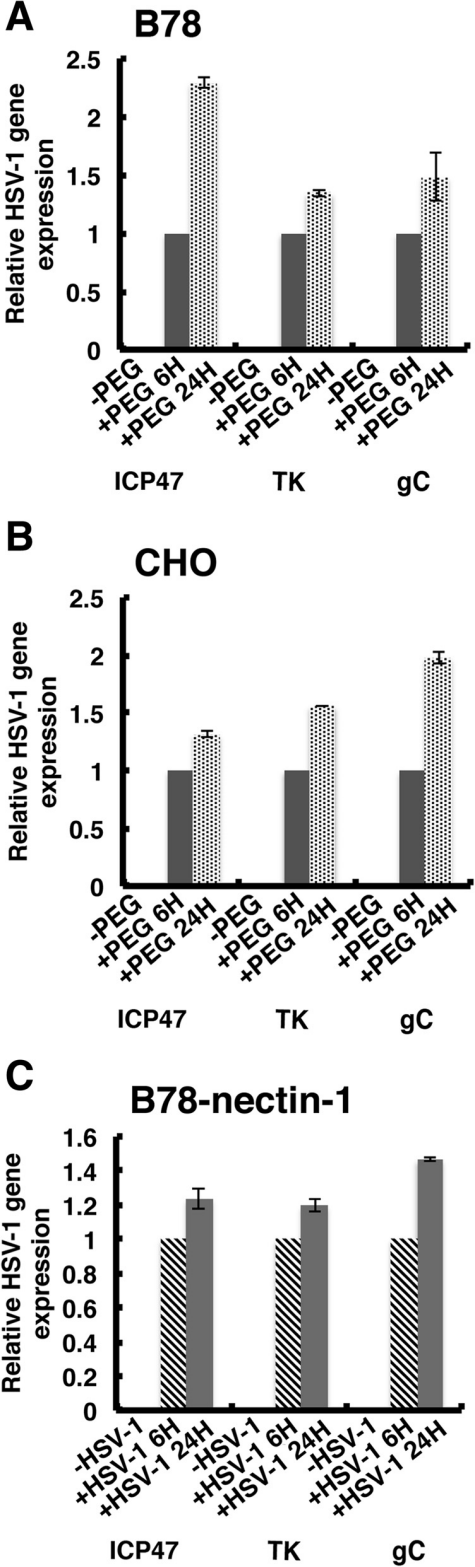
PEG-mediated entry leads to HSV-1 gene expression. At 6 or 24 h following either PEG treatment of HSV-1 bound to (**a**) B78 or (**b**) CHO cells or (**c**) following HSV-1 infection of B78-nectin-1 cells, RNA was extracted with TRIzol (Ambion). Following DNase treatment, first-strand cDNA was synthesized from 1.75 micrograms of total RNA using SuperScript VILO (Invitrogen). Equal volumes of cDNA were used to quantify HSV-1 gene ICP27, thymidine kinase (TK) and gC transcripts with Sso Advanced Sybr Green SuperMix (Bio-Rad), using a Bio-Rad CFX96 Real Time System. The quantity of HSV-1 mRNAs, normalized to cellular GAPDH, is shown relative to mRNA from 6 h post-PEG fusion

To explore how far infection progresses following PEG fusion of HSV-1 with B78 and CHO cells, viral gene expression and replication were measured by reverse transcriptase-qPCR with the indicated primers (Table 1). PEG-mediated entry of HSV-1 into B78 and CHO cells resulted in detectable expression of HSV-1 mRNAs representative of the immediate early (ICP27), early (thymidine kinase), and late (gC) gene classes (Fig. 3a and b). Detection of these specific proteins was not attempted. If viral proteins are indeed synthesized in PEG-treated CHO cells, the levels are likely too low to be detected by the antigen staining in Fig. 2. However, HSV-induced beta-galactosidase is clearly produced in these cells (Fig. 1).

**Table 1:** Primers used in this study

Gene	Primer sequence (5′- 3′)
HSV-1 ICP22	GAGTTTGGGGAGTTTG
	GGCAGGCGGTGGAGAA
HSV-1 ICP47	ACCGCTTCCTGCTCGT
	ACGCCCCCTTTTATTG
HSV-1 TK^a^	TCGGTCACGGCATAAGGC
	CAGCAAGAAGCCACGGAAGT
HSV-1 gC^a^	GTCCACCCTGCCCATTTC
	CGGACGACGTACACGATTGC
Mouse GAPDH	CGACTTCAACAGCAACTCCCACTCTTCC
	TGGGTGGTCCAGGGTTTCTTACTCCTT

^a^Primers modified from reference [76] to remove dimers and hairpins

**Fig. 4:**
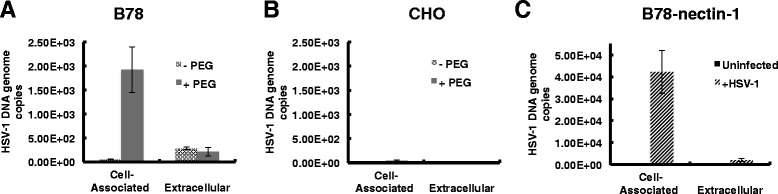
Detection of HSV-1 replication following PEG fusion. At 6 or 24 h following PEG treatment of HSV-1 bound to (**a**) B78 or (**b**) CHO cells (MOI of 10), DNA was extracted from cells with PureGene Kit (Qiagen) or from supernatant (Extracellular) with QIAamp DNA Blood Mini Kit (Qiagen). A standard curve for the assay was generated using known numbers of copies of a plasmid containing the HSV-1 ICP22 coding region diluted in glycogen. (**c**) Permissive control B78-nectin-1 cells were infected with HSV-1 for 24 h and processed as in panels **a** and **b**. 2.18E + 03 HSV-1 DNA genome copies were detected in the extracellular sample

PEG fusion of HSV-1 with B78 cells resulted in de novo synthesized viral genomic DNA as detected by qPCR (Fig. 4a). However, when an MOI as high as 30 was tested, extracellular progeny virions above the input inoculum were not detected. PEG-induced HSV-1 entry into CHO cells did not result in detectable viral DNA replication (Fig. 4b). CHO cells expressing the receptor HVEM are reportedly permissive for viral replication [39]. PEG-mediated entry into CHO cells at higher MOI might result in detectable HSV-1 replication. Likewise higher MOI in B78 cells might result in egress of progeny virions. HSV-1 infection of the permissive control cell line, B78-nectin-1 (MOI of 10), resulted in detectable viral DNA both inside the infected cell and in the extracellular medium (Fig. 4c). This is consistent with the ability of B78-nectin-1 cells to support HSV-1 plaque formation. Alternatively, nectin-1 may somehow rescue a post-replication defect in HSV-infected B78 cells. Thus, PEG fusion leads to successful entry, albeit at lower efficiency than nectin-1-mediated endocytosis, consistent with the results from Fig. 1. Together the results suggest that direct deposit of the nucleocapsid into the cytosol at the plasma membrane of CHO or B78 cells results in successful entry leading to viral gene expression or replication, respectively.

**Fig. 5:**
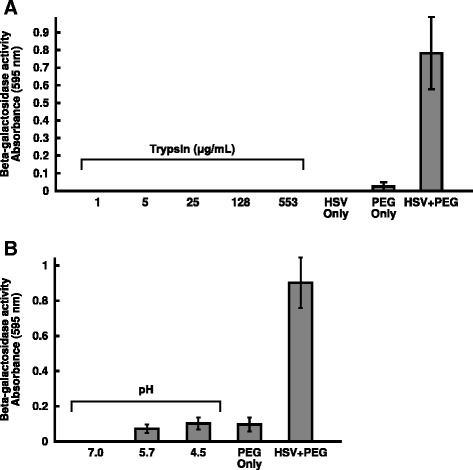
Inability of protease or low pH treatments alone to trigger fusion with the plasma membrane of entry-resistant cells. HSV-1 KOS was bound to B78 cells (MOI of 10) for 2 h at 4 °C. **a** Confluent cell monolayers were chilled at 4 °C and washed with ice-cold PBS. HSV-1 strain KOS in bicarbonate-free culture medium supplemented with 20 mM Hepes and 0.2 % BSA was added at an MOI of 10 for 2 h at 4 °C. Monolayers were rinsed with ice-cold PBS, and then PBS containing different concentrations of trypsin was added to cells for 25 min at 4 °C. Trypsinization was halted by addition of soybean trypsin inhibitor (8000-16,000 BAEE units; GIBCO-BRL) and 5 % fetal bovine serum for 15 min at room temperature. Complete cell culture medium was added, and cultures were incubated for 7 h at 37 °C. **b** Serum-free, bicarbonate-free culture medium with 0.2 % BSA and 5 mM (each) HEPES (Life Technologies), 2-(*N*-morpholino)ethanesulfonic acid (MES; Sigma), and sodium succinate (Sigma) was adjusted with HCl to achieve pHs ranging from 7.0 to 4.5 [69]. Confluent cell monolayers were chilled at 4 °C and washed with ice-cold PBS. HSV-1 strain KOS was added at an MOI of 10 for 2 h at 4 °C. Cells were washed with warm PBS. Warmed media adjusted to different pHs were added. Samples were incubated at 37 °C for 10 min. A pre-titrated amount of NaOH was added to return each sample to pH 7.4. Complete cell culture medium was added, and cultures were incubated for 7 h at 37 °C. Each value is the mean of quadruplicate determinations with standard deviation. Representative experiments of at least three independent experiments are shown

Cellular receptors that bind to gD are required for HSV entry, but it is not known whether they are sufficient. Since direct fusion of HSV-1 with receptor-negative cells results in entry without the need for endocytosis (Fig. 1), we were able to directly test potential triggers of virus-cell fusion. During entry of several viruses, cleavage of viral surface proteins by cellular endosomal proteases is a requirement for entry [4, 6, 54, 55]. In vitro protease treatment of viral particles can sometimes substitute for the cleavage during entry, and has been used for evidence that proteolysis is required for entry. We tested whether trypsin treatment of HSV-1 could trigger fusion with the surface of B78 cells as measured by beta-galactosidase expression. CHO cells were omitted from this analysis because of their sensitivity to even the lowest concentrations of trypsin tested (data not shown). HSV-1 was bound to the B78 plasma membrane at 4 °C, and then treated with increasing concentrations of trypsin. For limited proteolysis experiments, proteases are often added at 4 °C at which they are active ([56], unpublished data). Trypsin treatment under these conditions did not result in detectable viral entry (Fig. 5a). Importantly, control PEG treatment of cell-bound HSV-1 induced entry. In addition, inhibitors of cathepsins B and L do not block HSV entry [57]. We cannot discard the possibility that a distinct proteolytic event that is not mimicked by the trypsin treatments tested may yet play a role in HSV entry.

HSV requires intracellular low pH in a cell-specific manner [9, 11, 12, 22, 23]. Agents that alter endosomal pH such as bafilomycin A1 and ammonium chloride inhibit HSV entry into epithelial cells but not neurons [10]. HSV gB is triggered by low pH to undergo conformational changes [14, 58–62]. We have proposed that pH activates gB to trigger membrane fusion in part because the antigenic profile of low pH-treated gB is similar to that of the highly fusogenic gB present in fusion-from-without strains of HSV [63]. The entry requirement for low pH is shared by several herpesviruses, often in a cell-dependent manner [36, 64–66]. However, direct demonstration that acid pH triggers herpesviral fusion has remained elusive. We therefore asked whether low pH is able to directly trigger fusion of the HSV-1 envelope with the plasma membrane of a target cell. HSV-1 was bound to the B78 cell surface and then treated with different pHs. Treatment with pHs of 5.7 or 4.5 did not trigger HSV-1 entry above the background level detected when cells were treated with PEG in the absence of HSV (Fig. 5b). PEG treatment of cell-bound HSV-1 allowed entry and was included as a critical control. The current experiments suggest that proteolysis or low pH alone is not sufficient for entry. One explanation may be that a cascade of interactions with multiple cellular factors is necessary for fusion. The sequence of these interactions may also be critical.

Soluble, membrane-truncated, gD-receptors can also mediate HSV entry into B78 and CHO receptor-negative cells, but it is not known whether entry occurs at the plasma membrane [13, 67]. In fact, CHO cells endocytose herpes simplex virions and target them to a degradative pathway in the absence of known membrane-bound gD-receptors [11], so soluble gD-receptors may mediate entry via a CHO endosomal compartment. The current study cannot rule out that PEG treatment may somehow trigger fusion with the endosomal membrane. However, this is unlikely because PEG was added when virions were bound to the cell surface, and PEG causes fusion by complete mixing of the inner and outer leaflets of both membranes. To begin to address the mechanism of PEG-mediated fusion, we tested the ability of PEG to rescue the infectivity of cell-bound HSV-1 that was inactivated by sodium citrate buffer (pH 3.0). Low pH has long been known to inactivate HSV, but the mechanism of inactivation is not known [9, 59, 68–70]. PEG failed to rescue the inactivated virions as measured by beta-galactosidase reporter expression for reasons that are not clear (data not shown). Interestingly, this contrasts with the PEG rescue of HSV-1 mutants that lack either gD or gB [46, 47]. One explanation of our results is that PEG substitutes for a specific step(s) in the HSV fusion process and that PEG works together with the viral envelope to mediate full fusion. Under the conditions tested, low pH or trypsin were not capable of triggering HSV-1 fusion. This may suggest that neither is sufficient to substitute for the presence of a gD-receptor. It is possible that a combination of multiple host cell factors is necessary for HSV-1 fusion [3, 71–73]. Thus a sequential application of low pH, protease, and/or gD-receptors may be needed to mediate fusion of HSV-1 with the surface of receptor-negative cells. We demonstrate that B78 and CHO cells can be employed for such studies because PEG treatment of HSV-1 bound to cells likely results in fusion with the plasma membrane of these cells. Once identified, candidate molecules that permit HSV to fuse with the surface of B78 or CHO cells would be tested and confirmed in more physiologically relevant human cells.

## Conclusions

B78 cells and CHO cells, which typically endocytose HSV prior to viral penetration, are capable of supporting HSV-1 entry via direct penetration and may be utilized to screen for factors required for fusion of virions with the cell surface. PEG treatment can provide a needed control because it artificially fuses HSV with the host cell and results in HSV gene expression, and in the case of B78 cells, detectable viral replication. Compared to control entry mediated by PEG, the protease and pH conditions tested did not mediate penetration of HSV-1 at the cell surface of receptor-negative cells. One explanation is that a cascade of interactions with multiple cellular factors is likely necessary for fusion.

## Abbreviations

CHO: Chinese hamster ovary
gC: glycoprotein C
gD: glycoprotein D
HSV: Herpes simplex virus
HSV-1: Herpes simplex virus type 1
HSV-2: Herpes simplex virus type 2
HVEM: Herpes virus entry mediator
ICP22: Infected cell protein 22
ICP27: Infected cell protein 27
MOI: Multiplicity of infection
PBS: Phosphate buffered saline
PCR: Polymerase chain reaction
PEG: Polyethylene glycol
